# Drivers of apoplastic freezing in gymnosperm and angiosperm branches

**DOI:** 10.1002/ece3.3665

**Published:** 2017-11-28

**Authors:** Anna Lintunen, Stefan Mayr, Yann Salmon, Hervé Cochard, Teemu Hölttä

**Affiliations:** ^1^ Department of Forest Sciences University of Helsinki Helsinki Finland; ^2^ Department of Physics University of Helsinki Helsinki Finland; ^3^ Institute of Botany University of Innsbruck Innsbruck Austria; ^4^ University of Clermont‐Auvergne Clermont‐Ferrand France

**Keywords:** freeze–thaw cycle, freezing stress, ice nucleation, ice propagation, water content, winter embolism, winter tolerance

## Abstract

It is not well understood what determines the degree of supercooling of apoplastic sap in trees, although it determines the number and duration of annual freeze–thaw cycles in a given environment. We studied the linkage between apoplastic ice nucleation temperature, tree water status, and conduit size. We used branches of 10 gymnosperms and 16 angiosperms collected from an arboretum in Helsinki (Finland) in winter and spring. Branches with lower relative water content froze at lower temperatures, and branch water content was lower in winter than in spring. A bench drying experiment with *Picea abies* confirmed that decreasing branch water potential decreases apoplastic ice nucleation temperature. The studied angiosperms froze on average 2.0 and 1.8°C closer to zero Celsius than the studied gymnosperms during winter and spring, respectively. This was caused by higher relative water content in angiosperms; when branches were saturated with water, apoplastic ice nucleation temperature of gymnosperms increased to slightly higher temperature than that of angiosperms. Apoplastic ice nucleation temperature in sampled branches was positively correlated with xylem conduit diameter as shown before, but saturating the branches removed the correlation. Decrease in ice nucleation temperature decreased the duration of freezing, which could have an effect on winter embolism formation via the time available for gas escape during ice propagation. The apoplastic ice nucleation temperature varied not only between branches but also within a branch between consecutive freeze–thaw cycles demonstrating the stochastic nature of ice nucleation.

## 1 INTRODUCTION

Boreal and alpine trees experience frequent freeze–thaw cycles annually. Water in tree xylem and other apoplastic (i.e., outside living cells) spaces inevitably freezes when temperature drops a few degrees below zero. Freezing of apoplastic water causes two kinds of consequences for trees. First, gases dissolved in liquid water form bubbles during freezing, and these bubbles are at risk of expanding during thawing and impairing xylem transport by breaking the water column in embolism formation (Charrier et al., 2014; Sperry & Sullivan, 1992; Utsumi, Sano, Funada, Fujikawa, & Ohtani, 1999). Second, apoplastic freezing may induce symplastic freezing and further cellular damage (Charrier, Poirier, Bonhomme, Lacointe, & Améglio, 2013; Mazur, 1984; Ristic & Ashworth, 1993) if the living cells are not winter hardy and/or if the temperature of the apoplastic ice decreases very much.

The ecological significance of the apoplastic ice nucleation temperature to plant productivity and survival worldwide is likely less important in comparison with symplastic ice nucleation temperature that has been studied more extensively (e.g., Quamme, Weiser, & Stushnoff, 1973; Sakai and Larcher 1987; Malone & Ashworth, 1991; Lindstrom, Anisko, & Dirr, 1995; Taschler, Beikircher, & Neuner, 2004; Strimbeck, Schaberg, Fossdal, Schröder, & Kjellsen, 2015). However, a low apoplastic freezing temperature reduces the number of freeze–thaw cycles that a tree experiences during winter. For example, Lintunen, Hölttä, and Kulmala (2013) calculated that if the apoplast of a tree freezes at −10°C compared to a tree that freezes at −1°C, the number of freeze–thaw cycles is reduced by 85% and the total duration of frozen period by 50% in Southern Finland over a 15‐year‐long follow‐up period. An increase in the amount of freeze–thaw cycles accumulates the formation of winter embolisms (Lemoine, Granier, & Cochard, 1999; Mayr, Cochard, Améglio, & Kikuta, 2007; Mayr, Gruber, & Bauer, 2003; Mayr & Zublasing, 2010; Sparks & Black, 2000; Sparks, Campbell, & Black, 2001; Sperry, Nichols, Sullivan, & Eastlack, 1994) and cell damage (Augspurger, 2009; Lenz, Hoch, Vitasse, & Körner, 2013). Additionally, low ice nucleation temperatures allow evergreens to photosynthesize on winter days when air temperature is slightly below 0°C (Lindfors et al., 2015; Sevanto et al., 2006).

Although the apoplastic ice nucleation temperature determines the number and duration of annual freeze–thaw cycles in trees, it is not well understood what factors affect it. Most of the apoplastic water is located inside xylem conduits, but smaller amounts exist in, for example, intercellular gaps and cell walls. Water inside the xylem conduits is typically in a unique thermodynamic state in the sense that it is metastable to two directions before it freezes: It is simultaneously supercooled and under tension (Caupin, 2015; Debenedetti, 1996). The average time water may remain in its metastable liquid state is determined by the degree of supercooling and the properties of the heterogeneous nucleators it encounters (Wilson, Heneghan, & Haymet, 2003). Also the activity of water, that is, water potential, has been found to affect probability of heterogeneous ice nucleation in the atmosphere but also in some biological systems (Knopf & Alpert, 2013; Koop, Luo, Tsias, & Peter, 2000; Koop & Zobrista, 2009). Ice nucleators are needed as surface to catalyze ice nucleation at temperatures above the homogeneous ice nucleation temperature, which is approximately −39°C for water (Debenedetti, 1996). The osmotic concentration of apoplastic sap in tree branches and stems is so close to that of pure water that it decreases the equilibrium freezing temperature only by a fraction of a Celsius degree (e.g., Siebrecht, Herdel, Schurr, & Tischner, 2003), and thus, apoplastic ice nucleation temperature in these tree parts is mainly determined by supercooling.

Lintunen et al. (2013) found, when comparing samples of various species and tree parts, that apoplastic ice nucleation temperature varied with conduit size being higher in angiosperms than in gymnosperms, and hypothesized that conduit size would determine the size of the ice nucleators. However, in addition to the nature of ice nucleators, tree water status has been shown to affect apoplastic ice nucleation temperature of leaves and stems (Arias, Scholz, Goldstein, & Bucci, 2017; Carter, Brennan, & Wisniewski, 2001; Gusta, Wisniewski, Nesbitt, & Gusta, 2004; Medeiros & Pockman, 2011; Rada, Goldstein, Azocar, & Torres, 1987; Sperling, Secchi, Godfrey, & Zwieniecki, 2017; Wisniewski, Gusta, Fuller, & Karlson, 2009). Arias et al. (2017) showed that during winter acclimation of *Olea europaea* L., there was a decrease in stem water content combined with a decrease in apoplastic ice nucleation temperature. Also results by Carter et al. (2001), Medeiros and Pockman (2011) and Sperling et al. (2017) showed that the mean apoplastic ice nucleation temperature of *Ribes nigrum, Larrea tridentata and Pistacia integerrima*, respectively, was lower in dry stems in comparison with well‐watered stems.

The aim of this study was to analyze the linkage between apoplastic ice nucleation temperature and xylem water status in branches of 10 conifers and 16 angiosperm species in winter and in spring. To further analyze the linkage, we manipulated xylem water status in branches of Alpine *Picea abies*. We also analyzed the previously found correlation between conduit diameter and apoplastic ice nucleation temperature in fresh and saturated branches to understand the role of branch water status. Finally, we analyzed the linkage between apoplastic ice nucleation temperature and freezing dynamics and the role of stochasticity in the apoplastic ice nucleation of trees.

We hypothesized that (i) the decrease in branch water status lowers the apoplastic ice nucleation temperature across all studied species, (ii) branch water content and ice nucleation temperature are lower in winter than in spring and in conifers than in angiosperms, (iii) apoplastic ice nucleation temperature increases with increasing conduit diameter in both fresh and saturated branches, and (iv) the time required for apoplastic freezing decreases with decreasing ice nucleation temperature.

## 2 MATERIAL AND METHODS

### 2.1 Branch material

We collected altogether three sets of branches. The first two sets were collected to compare branch water status, ice nucleation temperature and exotherm duration, and magnitude between various tree species and between winter and early spring. The first sample set of winter‐acclimated branches was collected from an arboretum in Helsinki (Finland; 60°13′N; 25°00′E; 10 m.a.s.l.) in February 2016. The minimum and maximum air temperatures were −5.4°C and +9.7°C, −23.8°C and +4.4°C, and −8.4°C and +3.5°C in December, January, and February prior to the sampling, respectively. The sample set consisted of 10 gymnosperms and 16 angiosperms (see Table 1). The species were selected to represent a wide range of conduit sizes, and they represent boreal, sub‐boreal, and temperate species. The trees have been planted in 1970–1990 and have thus been acclimated to boreal climate for decades. We collected five branches from each tree species, from five different tree individuals whenever possible. The sampled branch segments were cut in air to a fixed length of 12 cm, their average diameter under bark was 5.4 mm (3.6–7.7 mm), and they were collected approximately at the same distance from the branch apex (the branches were of various lengths). The segments were without side twigs and needles with few exceptions, in which cases the side twigs were cut and needles were left intact. The branches were wrapped in plastic bags before cutting and immediately after cutting to avoid evaporation and brought to laboratory. All branches were collected on the same day between 10 am and 14 pm.

**Table 1:** Xylem ice nucleation temperature and relative branch water content of the sampled tree species in fresh (WF) and saturated (WS) status in winter and in fresh status in spring (SF)

	Ice nucleation temperature, °C			Relative water content		Max. conduit
	WF *n* = 15	WS*n* = 15	SF*n* = 15	WF*n* = 5	SF*n* = 3–5	*D*, μm*n* = 3
Angiosperms						
*Acer campestre*	−5.1 ± 0.4	−5.0 ± 0.1	–	0.95 ± 0.01	–	35.5 ± 4.6
*Acer platanoides*	−6.1 ± 0.3	−5.3 ± 0.1	–	0.90 ± 0.01	0.94 ± 0.00	48.0 ± 4.8
*Acer pseudoplatanus*	−5.8 ± 0.2	−5.1 ± 0.2	−4.5 ± 0.2	1.00 ± 0.01	0.99 ± 0.01	54.9 ± 7.0
*Alnus glutinosa*	−5.1 ± 0.2	−4.7 ± 0.2	−4.9 ± 0.2	0.94 ± 0.01	0.94 ± 0.00	50.2 ± 7.9
*Betula pendula*	−6.2 ± 0.3	−5.1 ± 0.4	−5.3 ± 0.2	0.91 ± 0.01	0.98 ± 0.01	56.3 ± 6.5
*Carpinus betulus*	−4.8 ± 0.3	−3.8 ± 0.1	–	0.95 ± 0.00	0.97 ± 0.00	55.0 ± 7.8
*Corylus avellana*	−5.0 ± 0.3	−5.0 ± 0.3	–	0.81 ± 0.01	0.88 ± 0.00	47.5 ± 10.1
*Crataegus monogyna*	−4.8 ± 0.3	−4.2 ± 0.1	−4.7 ± 0.4	0.91 ± 0.01	0.95 ± 0.00	39.6 ± 8.6
*Euonymus europaeus*	−5.4 ± 0.3	−4.5 ± 0.2	–	0.88 ± 0.01	0.91 ± 0.01	40.0 ± 9.2
*Populus alba*	−5.6 ± 0.3	−4.9 ± 0.2	–	0.92 ± 0.01	–	64.6 ± 7.6
*Populus nigra*	−6.7 ± 0.3	−4.7 ± 0.2	–	0.90 ± 0.01	0.91 ± 0.01	35.0 ± 6.7
*Populus tremula*	−5.5 ± 0.2	−4.5 ± 0.1	−5.0 ± 0.2	0.95 ± 0.00	0.96 ± 0.00	49.9 ± 5.9
*Prunus domestica*	−5.4 ± 0.3	−5.0 ± 0.1	–	0.98 ± 0.01	0.88 ± 0.01	67.9 ± 12.8
*Prunus padus*	−4.6 ± 0.3	−4.4 ± 0.1	–	0.86 ± 0.01	0.89 ± 0.03	51.2 ± 8.2
*Sorbus aucuparia*	−4.9 ± 0.1	−4.7 ± 0.1	–	0.95 ± 0.01	0.92 ± 0.01	39.5 ± 7.8
*Tilia platyphyllos*	−5.0 ± 0.3	−4.5 ± 0.2	–	0.87 ± 0.01	0.90 ± 0.01	78.4 ± 20.1
Gymnosperms						
*Abies grandis*	−4.5 ± 0.1	−4.5 ± 0.1	−6.3 ± 0.3	0.82 ± 0.01	0.83 ± 0.01	24.5 ± 5.9
*Juniperus communis*	−7.7 ± 0.3	−4.1 ± 0.1	−6.6 ± 0.1	0.75 ± 0.01	0.81 ± 0.02	22.0 ± 3.7
*Larix decidua*	−6.5 ± 0.1	−3.0 ± 0.2	−4.7 ± 0.1	0.72 ± 0.02	0.76 ± 0.01	24.3 ± 4.4
*Picea abies*	−7.7 ± 0.4	−4.0 ± 0.3	−5.7 ± 0.4	0.87 ± 0.00	0.90 ± 0.01	23.1 ± 4.7
*Picea engelmannii*	−7.5 ± 0.3	−3.5 ± 0.2	–	0.74 ± 0.04	0.84 ± 0.01	24.9 ± 5.0
*Pinus cembra*	−7.3 ± 0.2	−4.4 ± 0.1	−5.7 ± 0.2	0.82 ± 0.01	0.84 ± 0.01	23.1 ± 3.9
*Pinus contorta*	−9.2 ± 0.3	−4.4 ± 0.1	–	0.79 ± 0.00	0.81 ± 0.01	19.9 ± 4.0
*Pinus mugo*	−7.6 ± 0.3	−4.0 ± 0.3	–	0.79 ± 0.02	0.82 ± 0.01	23.7 ± 5.6
*Pinus sylvestris*	−8.4 ± 0.3	−6.2 ± 0.3	−8.3 ± 0.3	0.86 ± 0.01	0.93 ± 0.01	22.3 ± 2.8
*Taxus baccata*	−7.5 ± 0.3	−4.4 ± 0.2	–	0.86 ± 0.01	0.85 ± 0.00	20.4 ± 4.1

Average ± standard error within species is shown. Ice nucleation experiments in spring were only conducted for a subsample of 11 species.

The second set of branches was collected following the same protocol from the same species at the same location in the end of April 2016 after the thawing of the soil, but before budburst, when their water content can be expected to be higher than in the winter time (Table 1). In this spring sampling, three branches of all species (except two—see Table 1) were collected to measure their relative water content (see below), and five branches were collected from a subsample of 11 species for freezing experiments (Table 1, see below). The minimum and maximum air temperatures were −8.1°C and +9.2°C, and −1.3°C and +11.1°C for March and April prior to the sampling, respectively.

The third set of branches was collected to make an in‐depth comparison between ice nucleation temperature and manipulated water status of branches. Branches of *Picea abies* (L. Karst) were collected in May 2015 near the timberline in Praxmar (Tyrol, Austria; 47°09′N; 11°07′E; 1680 m a.s.l.). Trees were situated in an open *Picea abies* and *Pinus cembra* (L.) stand and were up to 3.5 m in height. Altogether, 42 branches of approximated length of 50 cm and width of 8 to 12 mm were collected on rainy days, wrapped in plastic bags, and transported to the laboratory.

### 2.2 Freezing experiments

**Figure 1:**
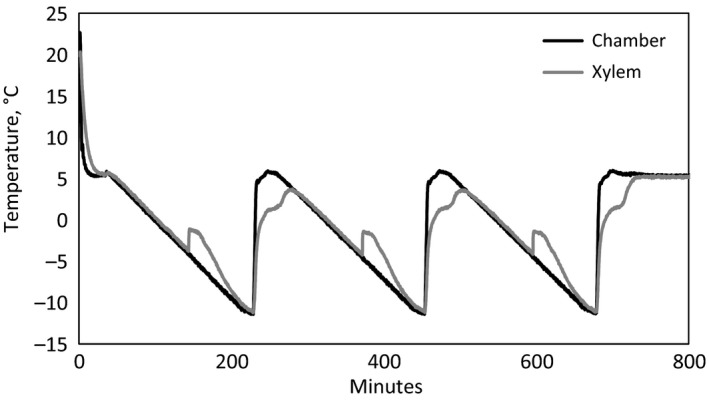
An example of the freezing experiments carried out with a branch of *Populus alba*. Freezing exotherms can be seen as increase in xylem temperature

The first two sets of branches were recut in air from both ends in the laboratory to fixed 8 cm length, and their fresh weight was measured. Cutting the segments inevitably emptied the cut‐open conduits from water, but further evaporation from the branches was prevented by keeping them in separate plastic bags through the whole experiment. As ice nucleation is an inherently stochastic process and even in two identical systems nucleation will occur at different times (Vali, 2014), all branches were exposed to three freeze–thaw cycles in a test chamber (WK11—340/40, Weiss Umwelttechnik, Vienna, Austria). One cycle included (i) a decrease in chamber temperature from 5°C down to −12°C with a fixed rate of 5.7°C per hr, (ii) a constant temperature of −12°C for 15 min, (iii) a rapid increase in temperature up to +5°C, and (iv) a constant temperature of +5°C for 30 min before the next cycle (Figure 1). During the experiment, branch and chamber temperatures were measured with T‐type thermocouples and recorded every 10 s with a data logger (CR1000, Campbell Scientific Ltd., Loughborough, Leicestershire, UK) to identify the freezing exotherms (Figure 1), that is, sudden increase in xylem temperature in relation to chamber air temperature due to thermal energy released from the wood due to the phase change in water into ice (Burke, Gusta, Quamme, Weiser, & Li, 1976). The branch thermocouple was attached to the bark surface as the amount of heat released from the freezing of xylem is clearly detectable there and this way the surface of the branch remains intact.

To analyze freezing dynamics of the apoplast, we calculated the duration and magnitude of the exotherms. First, difference between chamber and branch temperature (*T*_diff_) was calculated at every measurement point and then averaged over *N* measurement points (*N* = 7 measurement points were used as a compromise as large N would filter out better but induce a loss of accuracy). The beginning of an exotherm was determined as the point in time when the derivative of *T*_diff_ (=*T*_diff(*i*)_ – *T*_diff(*i*‐1),_ *where i is the measurements point number)* was larger than a preset threshold value of 0.1°C. The end of an exotherm was determined as the point in time when *T*_diff_ returned below a value which it had at the time step just before the start of the exotherm (*T*_diff(0)_). The magnitude of an exotherm (K·s) was then calculated as the time integral of *T*_diff_ minus *T*_diff(0)._ There were five individual freeze–thaw cycles where the exotherm formation was interrupted by the consecutive thawing process; consequently, these repetitions were deleted when freezing dynamics were analyzed.

After the freezing experiment, the branches were saturated (or rehydrated) by immersion in water at atmospheric pressure for 24 hr and weighed. Then, the freeze–thaw cycles (see above) were repeated with the saturated branches. At the end of the experiment, the branches were dried in an oven for 48 hr at 65°C and weighed to determine the relative water content of the fresh samples. Relative water content (RWC) was calculated as:

(1) $$\text{RWC}\;=\;\left({\text{WC}}_{F}\;-\;{\text{WC}}_{D}\right)/\left({\text{WC}}_{S}\;-\;{\text{WC}}_{D}\right)$$

where WC_*F*_ is water content of a fresh sample, WC_*D*_ is water content of a dry sample, and WC_*S*_ is water content of a saturated sample.

The third set of branches, shoots collected from *Picea abies*, were recut under water at the base when brought to laboratory and saturated for at least 24 hr in a cooling room (4°C). Branches were then dehydrated on the bench for different periods to achieve different water potentials for subsets of 4 to 8 branches per water potential point. Water potential was measured with a Scholander pressure chamber (model 1000 pressure chamber, PMS Instruments Co., Corvallis, USA) on one end twig. The branches were cut at the base to a total length of 30–40 cm before the freezing experiment. Further evaporation from the branches was prevented by keeping them in separate plastic bags through the whole freezing experiment. The freezing experiments were performed in a test chamber (MK53, Binder, Tuttlingen, Germany). Copper constantan thermocouples were installed in the xylem ca. 10 cm from the base, and xylem temperatures were recorded with 1‐min intervals (CR10X, Campbell Scientific Ltd., Loughborough, Leicestershire, UK). The branches were exposed to one freeze–thaw cycle down to −12°C with fixed temperature decrease rate following a similar protocol as for the first two sets (Figure 1).

### 2.3 Anatomical measurements

A 3‐cm‐long subsample was collected from three randomly selected branches per species from the first set and stored in −20°C until analysis. Transverse section was prepared from each subsample either with a sledge microtome (ca. 100 μm thick, Reichert‐Jung Optische Werke AG, Vienna, Austria) or with a rotary microtome (ca. 16 μm thick, Leica RM2265, Leica Microsystems, Wetzlar, Germany) using a cryostage and stained with safranin. One image was taken from each section from the outermost growth ring with a SPOT Insight B/W digital camera (Diagnostic Instruments Inc., Sterling Heights, MI, USA) attached to an Olympus BH‐2 microscope. The images were taken so that both reaction wood and opposite wood were avoided. The images were analyzed with Image‐Pro Plus imaging software (Media Cybernetics Inc., Bethesda, MD, USA) to measure the lumen diameter of three largest conduits (visual estimation) in each sample. Lumen diameter was measured in two directions, and the average per species was calculated.

### 2.4 Statistical analysis

The statistical tests were based on linear mixed models and residual maximum‐likelihood method (the mixed procedure in Statistical Analysis Software SAS). As the structure of the data was hierarchical, species and branches were treated as random factors.

To test differences between model estimates for different groups treated as fixed class variables, Tukey–Kramer *t* test was used. Differences in apoplastic ice nucleation temperature and relative branch water content were tested between the following groups: angiosperms vs. gymnosperms, fresh vs. saturated branches, and branches collected in winter vs. spring. We also tested differences in the standard deviation of ice nucleation temperature within a branch and between branches of each species. The results for testing fixed class variables are reported with model *p*‐value and estimated least‐squares means together with 95% confidence intervals in brackets (95 CI ±) after the mean.

The tested models with continuous fixed variables were apoplastic ice nucleation temperature against relative branch water content in fresh branches, change in ice nucleation temperature against change in branch water content due to change from winter to spring or due to saturating the fresh branches, ice nucleation temperature against manipulated stem water potential in *Picea abies*, and ice nucleation temperature against maximum conduit diameter. We also tested the relationship between the magnitude and duration of an exotherm against ice nucleation temperature. The results for testing fixed continuous variables are reported with *p*‐value and coefficient of determination (*R* ^2^).

Apoplastic ice nucleation temperatures of all freeze–thaw cycles were included in those analyses, where apoplastic ice nucleation temperature was the only continuous variable. In analysis containing branch water content, apoplastic ice nucleation temperatures from three consecutive freeze–thaw cycles were averaged per branch. In the analysis of maximum conduit diameter and that of standard deviation of ice nucleation temperature, apoplastic ice nucleation temperatures were averaged per species.

## 3 RESULTS

Fresh branches of gymnosperms froze on average at 2.0°C lower temperature than the branches of studied angiosperms in winter and 1.8°C in spring. Also relative branch water content was significantly lower for gymnosperms than for angiosperms both during winter and spring (Table 2). When branches were saturated with water in the laboratory, the freezing temperature became 0.5°C higher for gymnosperms than for angiosperms (Tables 1 and 2).

**Table 2:** Xylem ice nucleation temperatures (°C) and relative water content of angiosperms and gymnosperms

	Ice nucleation temperature			Relative water content	
	Fresh status in winter***	Saturated status in winter*	Fresh status in spring***	Fresh status in winter***	Fresh status in spring***
Angiosperms	−5.4 ± 0.4	−4.7 ± 0.3	−5.1 ± 0.6	0.92 ± 0.02	0.93 ± 0.03
Gymnosperms	−7.4 ± 0.6	−4.2 ± 0.4	−6.9 ± 0.6	0.80 ± 0.03	0.84 ± 0.03

Results are shown as averages ± 95% confidence intervals, and stars indicate significant differences (***<.001; **<.01; *<.05).

**Figure 2:**
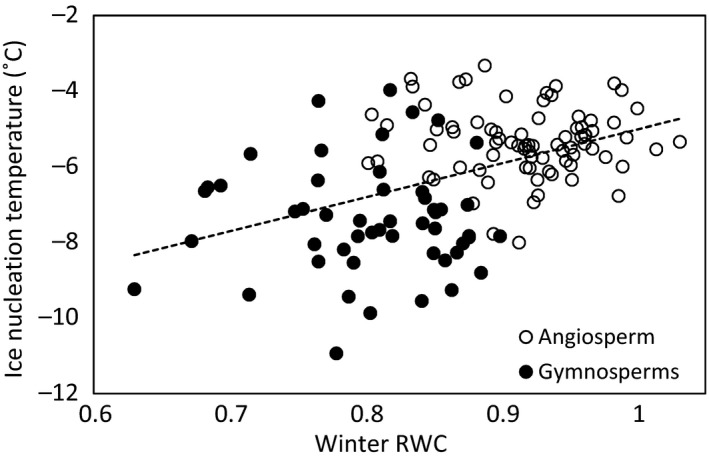
Relative branch water content in winter plotted against ice nucleation temperature. All branches are in fresh status. Linear fit is drawn with dashed line for all species (*y* = 9.0*x* – 14.0; *R* ^2^ = .21; *p* = .0001)

Mean relative branch water content was 0.90 (95% confidence interval ± 0.03) in spring and 0.87 (95% CI ± 0.03) in winter when data from angiosperms and gymnosperms were pooled together. The difference was significant (*p* < .0001), whereas difference in ice nucleation temperature between spring and winter was not significant. Relative branch water content explained ice nucleation temperature in winter (Figure 2, *p* = .0001) but not in spring.

**Figure 3:**
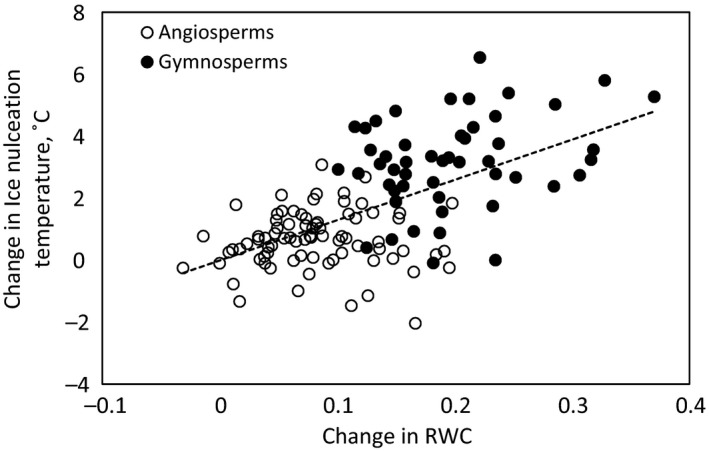
Change in relative branch water content (RWC) plotted against change in ice nucleation temperature between saturated and fresh branches in winter. Linear fit is drawn with dashed lines for all species (*y* = 13.0*x* – 0.009; *R* ^2^ = .37; *p* < .0001)

Ice nucleation temperature was higher in saturated branches than in fresh branches (p < .0001) harvested in winter: Mean ice nucleation temperature was −4.5°C (95% CI ± 0.3°C) in saturated status and −6.1°C (95% CI ± 0.3°C) in fresh status when data from angiosperms and gymnosperms were pooled together. Increase in relative branch water content was correlated with increase in ice nucleation temperature from fresh to saturated branch status (Figure 3, *p* < .0001).

**Figure 4:**
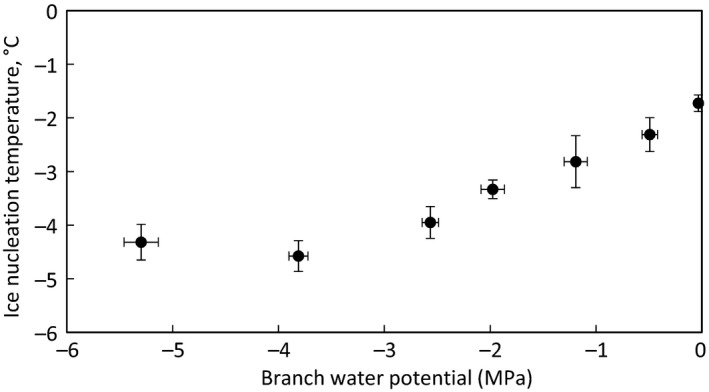
Average xylem ice nucleation temperature of *Picea abies* at different branch water potentials. Error bars show standard error, and sample size is 4–8 branches per water potential point

In *Picea abies*, ice nucleation temperature decreased with stem water potential linearly at ca. 0.75°C/MPa until −4.6°C (Figure 4). The minimum ice nucleation temperature of the branches was around −4.6°C, after which decreasing branch water potential did not further affect the ice nucleation temperature (Figure 4).

**Figure 5:**
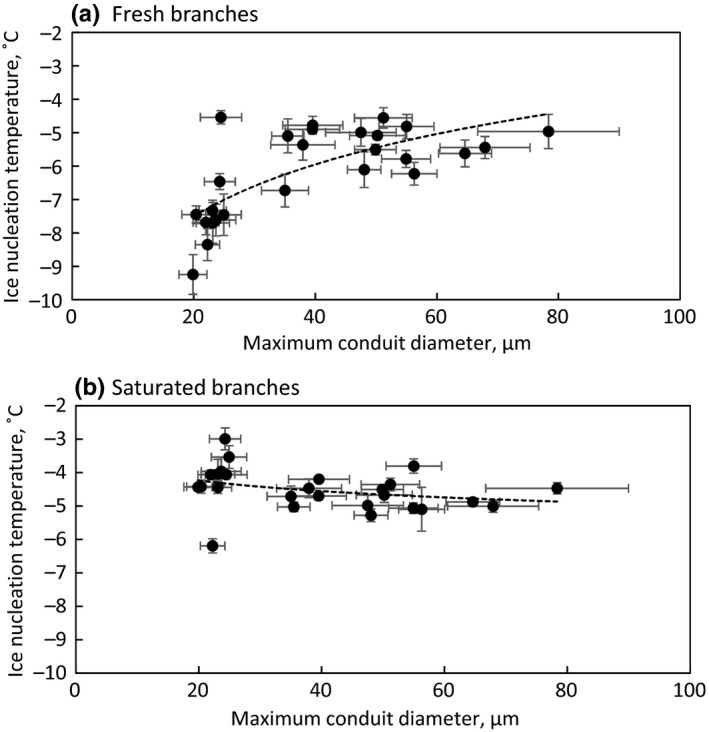
Species‐specific average of apoplastic ice nucleation temperature plotted against average maximum conduit diameter in fresh (a) and saturated (b) branches collected in winter. Error bars show standard error, and sample size is 5 for ice nucleation temperature and 18 for conduit diameter per species. Logarithmic fit is drawn with dashed lines for a (*y* = 2.3ln(*x*) – 14.3; *R* ^2^ = .53; *p* = .0086) and in b (*y* = −0.5ln(*x*) – 2.8; *R* ^2^ = .10; *p* > .05)

**Figure 6:**
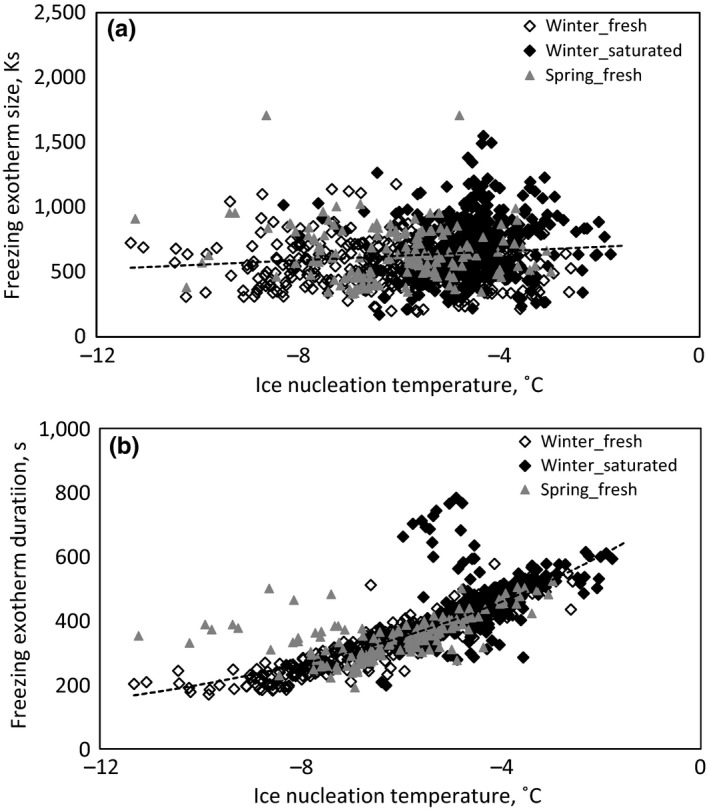
Magnitude (a) and duration (b) of freezing exotherm plotted against ice nucleation temperature during winter in fresh and saturated branches and during spring in fresh branches. Linear fit is drawn with dashed lines for all species in a (*y* = 17.3*x* + 729; *R* ^2^ = .015; *p* < .0001) and exponential fit in b (*y* = 793e^0.139*x*^; *R* ^2^ = .69; *p* < .0001)

Species‐specific mean ice nucleation temperature correlated logarithmically with conduit diameter in fresh branches collected in winter (Figure 5a, *p* < .0001) and in spring (*p* = .0044). No correlation was found between ice nucleation temperature and conduit diameter in saturated branches (Figure 5b). The magnitude (*p* < .0001) and duration (*p* < .0001) of the freezing exotherm were dependent on ice nucleation temperature, but the correlation was clearly stronger in terms of *R* ^2^ in the latter case (Figure 6).

**Figure 7:**
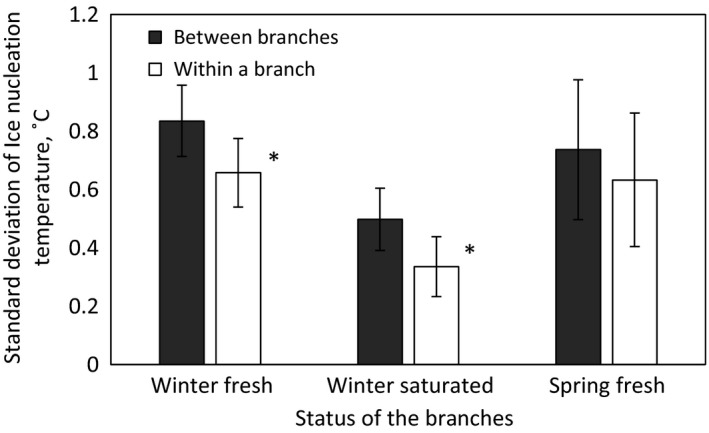
Average standard deviation of species‐specific ice nucleation temperature between branches and between freeze–thaw cycles within a branch is presented in different status of the branches. Error bars show 95% confidence intervals, and stars indicate significant differences (**<.01; *<.05)

Species‐specific standard deviation of ice nucleation temperature was 48% higher between branches than within a branch between the three consecutive freeze–thaw cycles in saturated winter status and 27% higher in fresh winter status (Figure 7). The difference was not significant when standard deviations of ice nucleation temperature were compared in fresh spring status of the branches (Figure 7).

## 4 DISCUSSION

The results show that a decrease in branch water status lowered the apoplastic ice nucleation temperature. Accordingly, the studied conifers had lower relative water content and thus lower apoplastic ice nucleation temperature than the studied angiosperms, and branches collected in winter had lower water content than branches collected in spring, but the difference in apoplastic ice nucleation temperature between winter and spring was not statistically significant. It was shown that apoplastic ice nucleation temperature increases with conduit diameter in fresh branches, but this correlation did not hold in saturated branches. The results show a strong correlation between apoplastic ice nucleation temperature and exotherm duration, whereas there was hardly any correlation between the apoplastic ice nucleation temperature and exotherm magnitude.

### 4.1 Apoplastic ice nucleation temperature vs. branch water status and conduit diameter

Branches with higher native water status had higher apoplastic ice nucleation temperature (Figure 2), and saturating the branches increased the apoplastic ice nucleation temperature (Figure 3b). Although the relation between branch water status and apoplastic ice nucleation temperature of trees has been shown also in previous studies (Arias et al., 2017; Carter et al., 2001; Goldstein, Meinzer, & Rada, 1994; Gusta et al., 2004; Medeiros & Pockman, 2011; Rada et al., 1987; Sperling et al., 2017; Wisniewski et al., 2009), this is the first study showing this relationship in the branches of variety of tree species. Also, the nature of the relationship between branch water status and apoplastic ice nucleation temperature has not been established. The effect of osmotic concentration of living cells on their equilibrium freezing temperature (i.e., melting point) has been well reported in the literature (George et al. 1982; Wisniewski, Gusta, & Neuner, 2014), but osmotic concentration of apoplastic sap in stems and branches is close to that of pure water (e.g., Siebrecht et al., 2003) and thus the equilibrium freezing temperature of the apoplast is close to 0°C. The decrease in water potential of the branches in our experiment was thus caused by a decrease in xylem water pressure, that is, increase in water tension, rather than by a decrease in osmotic pressure. In addition, it was the heterogeneous ice nucleation temperature, that is, the ability to supercool, rather than the equilibrium freezing temperature which was lowered, as a decrease in water pressure should affect the equilibrium freezing temperature only marginally and should actually increase it (Henderson & Speedy, 1987).

The decrease in ice nucleation temperature with decreasing branch water status was so strong that it cannot be simply explained with the effect of absolute water volume on ice nucleation probability (see Figures 3 and 4 in supplementary material in Lintunen et al., 2013); according to the classical nucleation theory, ice nucleation probability is dependent on the volume of water and the time exposed to temperatures below the melting point; that is, the time the sample is supercooled (Ashworth, Davis, & Anderson, 1985; Riechers, Wittbracht, Hütten, & Koop, 2013). The increased supercooling of apoplastic sap with decreasing branch water status can be approached with the concept of water activity. It has been empirically shown in atmospheric studies and in some biological systems such as larvae and insects that water activity plays an essential role in determining heterogeneous ice nucleation temperatures of liquids, together with ice nucleator properties (Knopf & Alpert, 2013; Koop & Zobrista, 2009; Koop et al., 2000). Water activity is defined as the ratio of water vapor pressure of solution to that of pure water in similar conditions, and it is affected by, for example, osmotic concentration and pressure (Nobel, 2005). Water potential can be calculated from water activity using the Kelvin equation (Nobel, 2005). It remains unknown whether direct link exists between water activity, water hydrogen bonding, and ice nucleation kinetics (Koop & Zobrista, 2009). The effect of water activity on ice nucleation temperature has been shown empirically by controlling solute concentration (Koop et al., 2000), but theoretically, the effect should be similar when water activity is determined by the water potential in the xylem. In the manipulation experiment with *Picea abies,* it was shown that the relationship between ice nucleation temperature and water potential is consistent and similar in shape and of comparable magnitude in relation to the previously reported relationships between ice nucleation temperature and water activity (Knopf & Alpert, 2013; Koop & Zobrista, 2009; Koop et al., 2000). Interestingly, the decrease in ice nucleation temperature levelled of below a water potential of ca. −4 MPa. This is the water potential at which 50% loss of conductivity (Mayr & Cochard, 2003; Mayr, Hacke, Schmid, Schwienbacher, & Gruber, 2006) are induced in *Picea abies*. A further decrease in water potential thus led to an increasing portion of air‐filled tracheids, and the freezing point of the remaining xylem water then was obviously independent from water potentials.

Apoplastic ice nucleation temperature increased with increasing conduit diameter in fresh branches as has been shown before (Lintunen et al., 2013). However, when the branches were saturated with water, the correlation between conduit diameter and ice nucleation temperature disappeared. This implies that conduit diameter does not explain apoplastic ice nucleation directly, but through tissue water content. Conduit diameter and branch water content were correlated between species in winter (*p* = .0006) and in spring (*p* = .0013) with species having larger conduits having also higher water content. This relationship can be largely explained by the difference between angiosperms and gymnosperms in both variables as such relationships did not exist if only one of the species groups was analyzed.

It is known that intact branches tend to freeze at higher temperatures than cut branches (Ashworth et al., 1985; Pramsohler, Hacker, & Neuner, 2012) because smaller tissue samples supercool to lower freezing temperatures (Burke et al., 1976). For example, measured apoplastic ice nucleation temperature for intact *Picea abies* and *Pinus sylvestris* saplings in the laboratory has been measured to be −4.5 and −3.3°C in February (Lintunen et al., 2014), which is 2.9 and 5.1°C higher than measured for detached branches of mature trees of the same species in the present study. However, the mechanism of decreasing apoplastic ice nucleation temperature with decreasing water content should be similar in intact and detached branches.

### 4.2 Apoplastic ice nucleation temperature and branch water status in winter and spring and in different species

Branch water content was lower in winter than in spring, but the difference was not sufficient to induce difference in the apoplastic ice nucleation temperature (Table 1). In contrast, Arias et al. (2017) showed that both leaf water potential and stem water content decreased in winter and induced decrease in the apoplastic ice nucleation temperature in *Olea europaea* L. It is possible that we did not catch the maximum water status after spring recovery for all species when sampling in late April, because different species refill in different times (Hacke & Sauter, 1996). However, to avoid confounding effect due to transpiration, the sampling had to be carried out before leaves burst in the angiosperms in early May. It is known that tree water content is typically lowered in winter (Charriera & Améglio, 2011; Mayr, Wolfschwenger, & Bauer, 2002; Mayr et al., 2006; Richards & Bliss, 1986; Sparks, Campbell, & Black, 2000; Sparks et al., 2001) and it has been connected to tree frost hardiness (Charrier et al., 2013; Charriera & Améglio, 2011; Medeiros & Pockman, 2011; Ögren, 1999). For example, Sperling et al. (2017) found that dehydrated *Pistacia integerrima* (J. L. Stewart ex Brandis) stems had more soluble sugars, higher proline levels, and less frost damage in the living cells than well‐watered trees after being exposed to a freeze–thaw cycle.

Ice nucleation temperature differed between angiosperms and gymnosperms mainly due to differences in branch water content: Gymnosperms froze at approximately 2°C lower temperature than angiosperms both in winter and in spring, but when branches were saturated with water, apoplastic ice nucleation temperature of gymnosperms increased to slightly higher temperature than that of angiosperms. It has been shown before that gymnosperms freeze at lower temperatures than angiosperms in fresh status (Lintunen et al., 2013). Lower relative water content in the studied gymnosperms in comparison with the studied angiosperms could be related to evergreen foliage in conifers. As transpiration from the needles continues in winter, but the transpired water cannot be replaced by water uptake from the cold soil, xylem water content can be expected to decrease in evergreens during winter especially in boreal and alpine regions. The difference in water status also existed in spring as the measurements were made before leaf burst in the angiosperms. This result is in agreement with Sparks et al. (2000) who observed lower water content in gymnosperms *Pinus contorta* (Dougl. ex Loud.) and *Larix occidentalis* (Nutt.) than in a diffuse porous angiosperm *Populus trichocarpa* (Torr. & A. Gray) during winter. Evergreens might profit from water potential‐induced lower ice nucleation temperatures as it allows to photosynthesize on winter days when air temperature is slightly below 0°C (Lindfors et al., 2015; Sevanto et al., 2006) and it decreases accumulation of winter embolism and cell damage by reducing the number of annual freeze–thaw cycles.

When branches were saturated with water, gymnosperms froze at slightly (0.5°C) higher temperature than angiosperms (Table 2). One possible explanation is the difference in the absolute volume of apoplastic water in saturated branches of these species groups. Ice nucleation probability increases with increasing water volume, and thus, freezing temperature is slightly higher in samples with higher apoplastic water volume (see figs. 3 and 4 in Supplementary Material in Lintunen et al., 2013). When branches of fixed size are saturated with water, conifers likely have higher apoplastic water volume as they have higher apoplast to symplast ratio in their branch volume (Spicer, 2014) and also lower wood density implying lower cell wall to lumen area ratio in their branch volume than angiosperms (Sperry, Hacke, & Pittermann, 2006).

We used relative branch water content instead of direct water potential measurement to link branch water status with apoplastic ice nucleation temperature in different species with the exception of the additional bench drying experiment conducted with *Picea abies*. Direct measurements of water potential would theoretically be better, because relative branch water content represents both apoplastic and symplastic water in the sample, and the share of symplastic tissue is larger in angiosperms than in gymnosperms (Spicer, 2014). This means that any possible water storage dynamics that would lead to changes in the share of water between apoplast and symplast can induce some variation in the ratio of branch water content and water potential and further in the ratio of branch water content and apoplastic freezing temperature between species. However, by measuring relative branch water content instead of water potential from the fresh branches, we avoided possible disturbance for the samples before freezing them as water potential measurement would have led to some removal of water from the sample and to relocation of water within the sample due to the technique itself. Within a species, the relationship between water potential and water content has been shown to be rather stable in woody tree parts especially at relatively high water contents (RWC > 0.60; Hellkvist, Richards, & Jarvis, 1974; Meinzer, James, Goldstein, & Woodruff, 2003; Turner, 1988; Tyree & Hammel, 1972) as measured in the present study.

Apoplastic ice nucleation temperature within a species varied more between branches than between freeze–thaw cycles within a branch. This difference was statistically significant only when branches were in fresh or saturated status in winter due to high variation in branch water content in spring. Within a branch, the variation in ice nucleation temperature between consecutive freeze–thaw cycles is caused by the stochastic nature of ice nucleation (Debenedetti, 1996; Vali, 2014). This means that ice nucleation temperature is different in each freezing event even without any measurement inaccuracy, and a normal distribution of freezing temperatures would be achieved if freeze–thaw cycles are repeated many enough times (Wilson et al., 2003). However, this kind of freezing precision is easier to achieve with abiotic materials that can go through numerous, fast freeze–thaw cycles without any effect on the material (Wilson et al., 2003). The three repetitive freeze–thaw cycles conducted in this study are still an improvement compared to earlier studies of apoplastic freezing in trees, because, to the best of our knowledge, they did not consider the stochasticity of the process (Arias et al., 2017; Carter et al., 2001; Medeiros & Pockman, 2011; Rada et al., 1987; Sperling et al., 2017). Additional variation in ice nucleation temperature between branches was caused by differences in, for example, water status, overall variability of branches (variation in compression/tension wood portions, density etc.), and possibly also by the composition of ice nucleators in the sap (Vali, 2014; Wisniewski et al., 2002).

### 4.3 Freezing dynamics

A clear correlation was found between ice nucleation temperature and the duration of a freezing exotherm, but not between ice nucleation temperature and the magnitude of the exotherm (Figure 6). Exotherm magnitude is linked to ice nucleation temperature via water volume and the degree of supercooling: The lower the branch water content, the lower the ice nucleation temperature is (based on the results of this study) and the higher the energy release due to higher supercooling (Burke et al., 1976). These effects act in reverse directions with respect to the magnitude of an exotherm and likely cancel each other out, hence the lack of observed correlation. The duration of an exotherm represents ice propagation velocity, and it is known from previous studies that low apoplastic ice nucleation temperature causes high ice propagation velocity in trees (Charrier et al., 2015; Hacker & Neuner, 2007; Kitaura, 1967; Langer, Sekerka, & Fujioka, 1978; Rauschenberger et al., 2013). Thus, it can be concluded that the clear correlation found between ice nucleation temperature and exotherm duration represents mainly linkage between ice nucleation temperature and ice propagation velocity.

It is likely that the direct effect of low ice nucleation temperature on winter embolism formation is such that it decreases the number of freeze–thaw cycles, which decreases the accumulation of winter embolism during winter (e.g., Mayr et al., 2003). But the indirect link of ice nucleation temperature on winter embolism formation via its linkage to xylem water content and exotherm duration is less clear. Winter embolism formation is determined by the size of gas bubbles formed in the ice during freezing and the water potential in xylem during thawing: the risk for the bubbles to expand and embolise the conduits during thawing increases with increasing bubble size and decreasing pressure (Ewers, 1985; Pittermann & Sperry, 2003, 2006). On the one hand, low xylem water content, which increases the risk of winter embolism formation (Davis, Sperry, & Hacke, 1999; Martínez‐Vilalta & Pockman, 2002; Mayr & Sperry, 2010; Wilson & Jackson, 2006), is linked to low ice nucleation temperature (Figs. 2, 3, 4). On the other hand, low ice nucleation temperature is linked to fast ice propagation velocity (Figure 6), and physical freezing experiments with thin ice sheets show that the faster the ice propagation velocity is the more and the smaller are the gas bubbles formed in the ice (Carte, 1961). This would decrease the risk of winter embolism formation (Mayr & Améglio, 2016; Pittermann & Sperry, 2006). Furthermore, fast ice propagation will shorten the time for the gases to escape from the freezing conduits, and hence, the total amount of gases trapped inside the ice and the risk for embolism formation might increase (Lintunen et al., 2014). Further studies are required to estimate the relevance of these, partly contrary effects of ice nucleation temperature on the formation of winter embolism.

## CONFLICT OF INTEREST

The authors have no conflicts of interests to declare.

## AUTHOR CONTRIBUTIONS

AL, TH, and YS collected the samples and made the laboratory work in Helsinki and SM in Austria. AL had the main responsibility in data analysis and writing the manuscript. All authors contributed in planning the experiment and writing the manuscript.
